# A 480-million-year-old parasitic spionid annelid

**DOI:** 10.1016/j.isci.2025.113721

**Published:** 2025-10-14

**Authors:** Karma Nanglu, Madeleine E. Waskom, Sarah R. Losso, Javier Ortega-Hernández

**Affiliations:** 1 Museum of Comparative Zoology and Department of Organismic and Evolutionary Biology, Harvard University, Cambridge, MA 02138, USA; 2 Department of Ecology and Evolutionary Biology, University of Toronto, 25 Willcocks St, Toronto, ON M5S 3B2, Canada; 3 Department of Earth and Planetary Sciences, University of California, Riverside, 900 University Avenue, Riverside, CA 92521, USA

**Keywords:** Natural sciences, Biological sciences, Paleobiology

## Abstract

The Paleozoic fossil record provides unique insights into the evolution of life history traits through the direct preservation of interspecific interactions in deep time. However, evidence of direct interactions between different species is relatively rare even among localities with exceptional soft-tissue preservation. Here we provide evidence of parasitic organisms from the Fezouata Shale biota of Morocco. Seven specimens of the bivalve mollusk *Babinka* show highly characteristic, question mark-shaped shell borings consistent with those produced by modern and fossil parasitic spionid polychetes. This suggests that the spionid polychetes, or polychetes with behavior consistent with spionids, were present in the Early Ordovician, a significant biostratigraphic shift in their temporal origins from their accepted Devonian occurrence. Many unique life history strategies which were significant components of the Fezouata Shale biota remain undiscovered, despite the high concentration of taxonomic attention on the site.

## Highlights

• The first evidence of parasitism from best preserved Ordovician fossil site, Fezouata
• Parasitic spionids occurred ∼100 million years older than previously known
• Life history strategy of these worms has remained stable for 480 million years

## Graphical abstract

**Figure:**
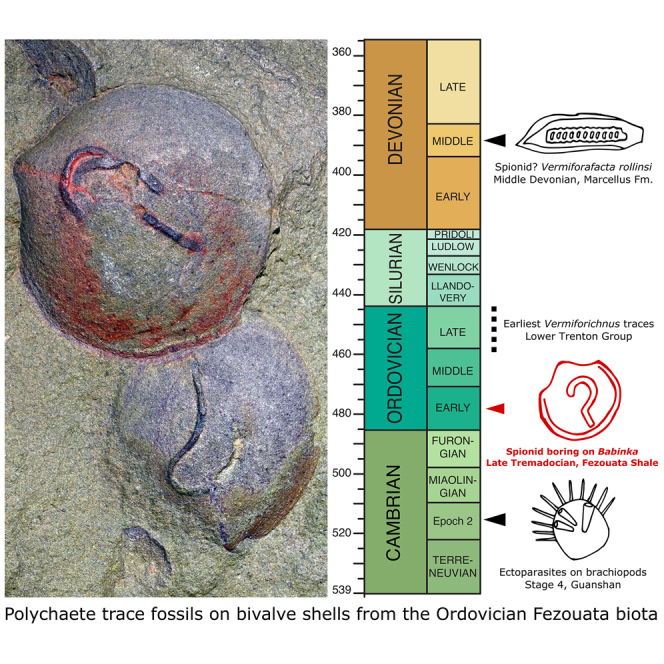

## Introduction

Evidence of parasitic ecological interactions in the fossil record is critical for understanding how the life history strategies of animals have evolved and to unveil the full complexity of the ecosystems in which they lived.^1^ Despite evidence for instances of ancient parasitism among broad taxonomic groups^2^ across the Phanerozoic, it is significantly rarer in sites of exceptional soft-tissue preservation.^1^^,^^3^^,^^4^^,^^5^ Here, we report an exceptionally preserved instance of a distinctive shell boring parasitic behavior between a spionid-like polychete worm and the bivalve mollusk *Babinka* from the Early Ordovician (ca. 480 million years ago) Fezouata Shale biota in Morocco. The discovery of this shell boring association carries three main implications for understanding the evolutionary history of parasitism. The morphology of the fossilized borings indicates behavioral complexity previously unknown from Paleozoic polychetes.^5^ The presence of boring traces in *Babinka* documents the first evidence of parasitic behavior from the Fezouata Shale biota,^6^ and the earliest instance of shell boring parasites on mollusks in the fossil record.^1^^,^^5^^,^^7^ Finally, the boring traces extend the earliest evidence for the polychete order Spionida into the late Tremadocian.^1^^,^^5^^,^^8^

## Results

### Specimens

**Figure 1:**
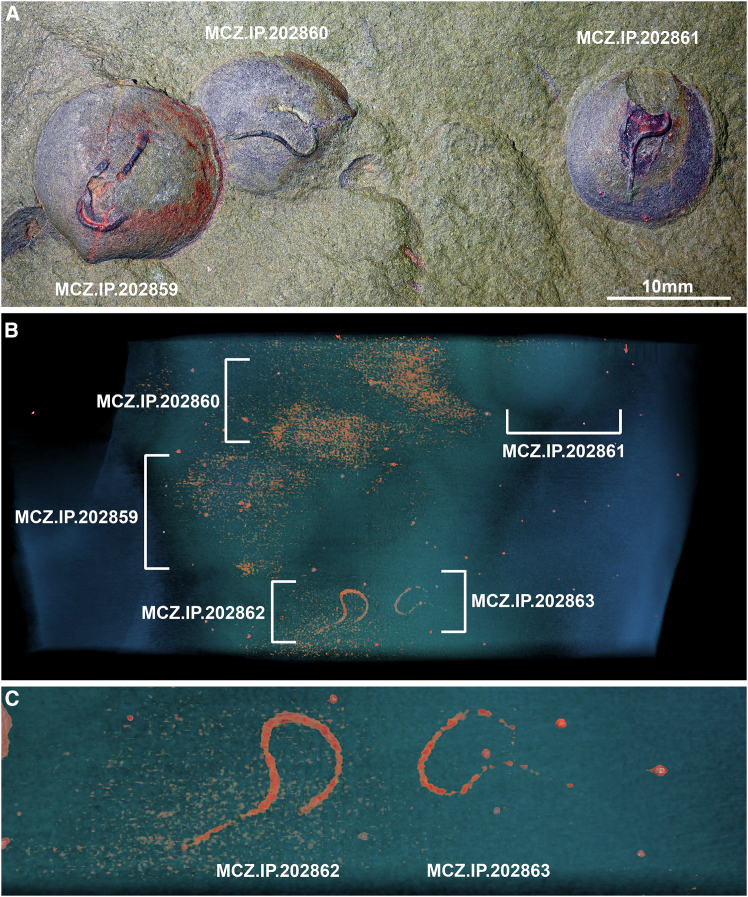
Fossiliferous slab with specimens of the bivalve mollusk *Babinka* from the Fezouata Shale biota (Early Ordovician) showing evidence of fossilized spionid-like borings (A) *Babinka* cluster; from left to right, MCZ.IP.202859, MCZ.IP.202860, and MCZ.IP.202861. (B) Tomographic reconstruction of fossiliferous slab showing additional evidence of spionid-like borings preserved as iron oxides within the rock matrix. (C) Magnification of spionid-like borings within rock matrix. MCZ.IP.202862 (left) and MCZ.IP.202863 (right).

**Figure 2:**
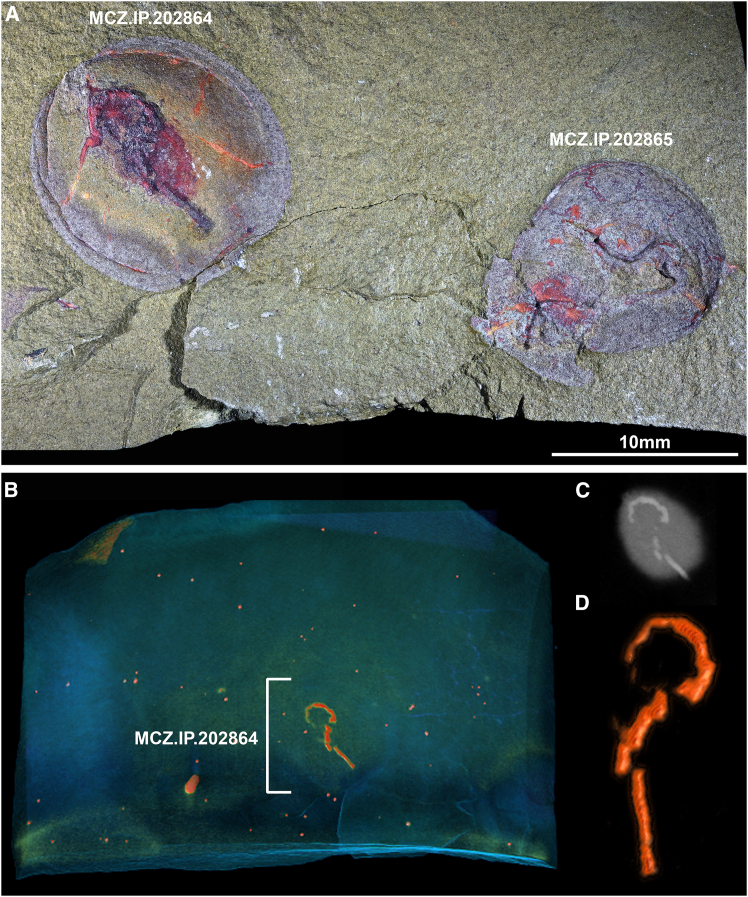
Specimens of the bivalve mollusk *Babinka* from the Fezouata Shale biota (Early Ordovician) showing evidence of fossilized spionid-like borings (A) From left to right, MCZ.IP.202864 and MCZ.IP.202865. (B) Tomographic reconstruction of fossiliferous slab spionid-like boring preserved as iron oxides in MCZ.IP.202864. (C) Tomographic section of boring specimen showing high density of iron oxides relative to aluminosilicate matrix. (D) Magnification of isolated spionid-like boring in MCZ.IP.202864.

The studied material includes 22 individuals of the bivalve *Babinka* from the Early Ordovician (late Tremadocian) Fezouata Shale deposited at the Invertebrate Paleontology collection of the Museum of Comparative Zoology (MCZ.IP) at Harvard University (Figures 1 and 2). These fossils constitute less than 1% of the roughly 9,000 Fezouata Shale individual specimens in the MCZ, making it among the rarest taxa in this collection. Mollusk bivalves are a relatively small proportion of the overall biodiversity of Fezouata Shale biota, being represented by nine genera.^9^ Although relative abundance data are not available for most Fezouata Shale collections globally, there appears to be spatiotemporal heterogeneity among the Fezouata localities such that bivalves may be rare in some sites but abundant in others.^9^

### Preservation

*Babinka* specimens are preserved as internal molds in fine-grained argillite matrix, with no evidence of the original biomineralized shell evidenced by the smooth texture and absence of shell external ornamentation such as growth lines (Figures 1A and 2A). The surface of the valve internal molds in seven specimens show three-dimensional horizontal trace fossils with a highly consistent appearance including a counter clock-wise loop oriented toward the valve’s dorsal hinge, and a straight tube oriented toward the commissure, that conveys the appearance of a stylized question mark (Figures 1A and 2A). The occurrence of the fossilized borings on the surface of the valve internal mold indicates that the boring would correspond to the internal (visceral) side of the shell during life. The trace fossils are replicated in iron oxides denser than the aluminosilicate matrix (Figures 1B, 1C, and 2D) and maintain the same width (ca. 0.5 mm) throughout their entire length. Micro-computed tomography reveals additional instances of shell boring traces within the rock matrix in one of the studied slabs thanks to their preservation through iron oxide infills and extremely distinctive morphology, even though the corresponding *Babinka* are entirely covered by sediments (Figure 1B). Some specimens feature simpler secondary traces that are less organized and extend throughout the valve surface (Figure 2A).

We interpret the presence of three-dimensional borings in *Babinka* specimens as a result of diagenetic events affecting the fossils at different time frames. The presence of traces replicated by three-dimensional iron oxides can be readily explained by the activity of sulfur-reducing bacteria feeding off of organic matter within the original borings in the shell, leading to the precipitation and accumulation of pyrite during early diagenesis. Indeed, pyritization of labile tissues is commonplace in the exceptional horizons within the Fezouata Shale.^10^ The original calcitic shell would have subsequently dissolved, most likely due to localized changes in acidity associated with low oxygen conditions conducive to exceptional preservation, leaving behind internal molds of *Babinka* with the pyritized spionid borings on top as expressed in the studied material. The loss of calcitic shells is a widespread phenomenon in the Fezouata Shale, affecting biomineralizing organisms such as mollusks^9^^,^^11^ and echinoderms.^12^^,^^13^ In this context, the pyritized borings would not have been affected by the loss of the calcitic shell of *Babinka*, producing the distinctive appearance of this material.

## Discussion

**Figure 3:**
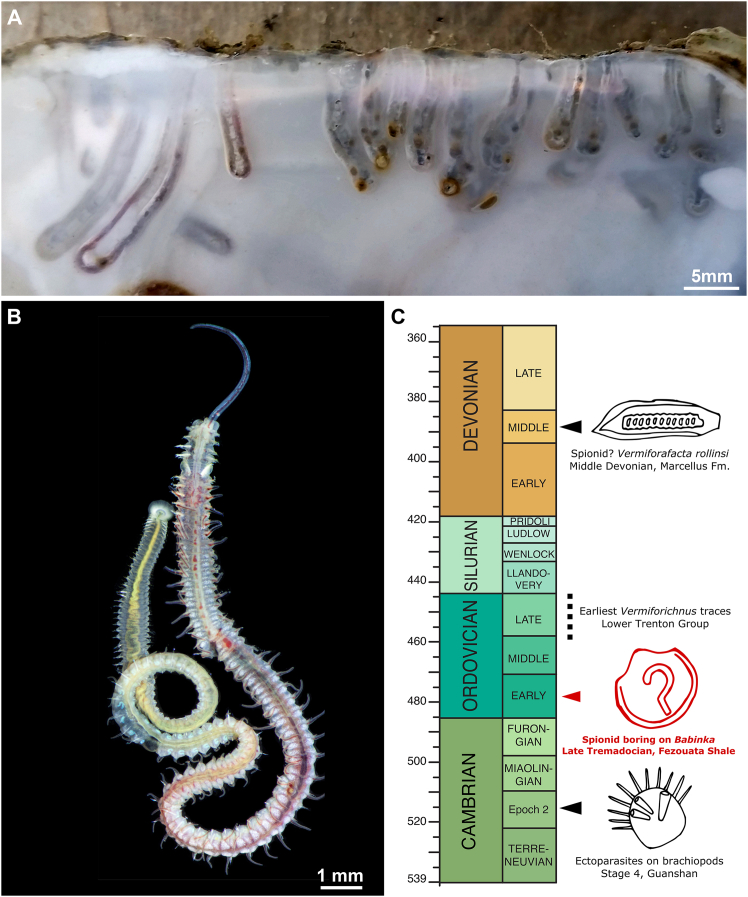
The extant spionid genus *Polydora* and biostratigraphic distribution of spionid-like borings during the Paleozoic (A) Polydorid U-shaped shell borings on Pacific oyster *Magallana gigas* (image courtesy of V. Radashevsky; see Radashevsky et al.^16^). (B) Adult of the spionid polychete *Polydora hoplura* (image courtesy of V. Radashevsky; see Radashevsky et al.^16^). (C) Simplified geological timescale of evidence for bivalve shell parasitism during the Paleozoic^1^^,^^5^^,^^19^; fossil traces in *Babinka* from the Fezouata Shale represent the earliest example of shell boring on bivalve mollusks to date, and the oldest record of spionid-like borings.

The morphology of the trace fossils on *Babinka* is congruent with the dwelling borings created by extant spionid polychetes in bivalve mollusk shells^14^ which are produced by the formation of U- or flask-like traces after penetrating a crevasse on the shell surface^5^^,^^14^ (Figures 3A and 3B). Borings produced by the spionid *Polydora* have a diversity of shapes,^15^ but typically have a recurved appearance that can be described in three broad categories: a tight U-shape where both boreholes are roughly parallel to each other^16^ (Figure 3A); a wider curve with more obtuse angles between each bend^17^; and a boring where a sharp initial bend leads to a recurved U-shape similar to a question mark.^14^ The trace fossils described herein are similar to the last of these morphotypes, consisting of a long straight boring followed by four counter-clockwise turns with obtuse angles between 114° and 144° (Table 1), except in the case of MCZ.IP.202864, which does not form a complete final loop (Figure 1B). This is in contrast to the U-shaped and widely curved morphotypes, the former of which consists of only two sharp angles (Table 1), and the latter lacks the consistent regular shape of the *Babinka* trace fossils. *Polydora* also creates characteristic blisters within their tubes that are subsequently filled with mud and feces.^16^ These mudblisters form as a part of the host response to infection,^18^ and one specimen (Figure 2A) shows similarity in three-dimensionality and position to mudblisters formed by *Polydora ecuadoriana* is the mangrove oyster *Crassostrea rhizophorae* (see Figure 6C in Radashevsky et al.^18^). These structures would allow us to more confidently identify the trace fossils on *Babinka* as a result of parasitism rather than a postmortem colonization of the shell. However, the similarities between these structures and modern mudblisters may be superficial and is only observed in one specimen out of seven, so caution is warranted in this interpretation. Additionally, we cannot definitively rule out the possibility that the trace fossils on *Babinka* we describe were created by another type of worm (polychete or otherwise) which evolved a similar boring behavior convergently. However, when we consider the similarities in overall morphology between the Ordovician borings and extant spionid borings in a variety of mollusk hosts, we would argue that an affinity within the family Spionidae (or, minimally, the order Spionida) is most likely.

**Table 1:** Morphology of spionid boring traces in fossil bivalve *Babinka* from the Ordovician Fezouata locality, with comparisons to extant spionid bivalve traces

Publication/specimen	Figure	Shape	Angle 1	Angle 2	Angle 3	Angle 4
MCZ.IP.202859	1A	question	123.477	113.641	141.459	129.013
MCZ.IP.202860	1C	question	144.339	140.615	126.66	142.212
MCZ.IP.202861	1C	question	123.477	113.641	141.459	129.013
MCZ.IP.202862	S1C	question	126.594	109.069	135.551	137.757
MCZ.IP.202863	S1C	question	125.512	120.042	134.465	134.488
MCZ.IP.202864	S2B	question	123.111	114.956	131.278	–
MCZ.IP.202865	S2A	question	129.242	115.863	147.319	129.936
Zottoli and Carriker 1974^14^	2	question	139.939	121.744	139.422	140.917
Sato-Okoshi et al. 2023^17^	7F	wide	99.884	149.717	–	–
Sato-Okoshi et al. 2023^17^	7F	wide	114.632	102.743	–	–
Sato-Okoshi et al. 2023^17^	7F	wide	132.357	120.39	87.739	–
Radashevsky et al. 2023^16^	4G	U shaped	102.885	110.583	–	–
Radashevsky et al. 2023^16^	4G	U shaped	105.542	96.144	–	–
Radashevsky et al. 2023^16^	4G	U shaped	87.842	87.762	–	–

The simpler traces observed in MCZ.IP.202864 (Figure 2A) may correspond with newer, less developed infections, as also observed in modern bivalves with polydiarosis that show parasitic boring morphotypes with variable width, extent, and shape within a single specimen.^16^ They may also represent separate infestations by multiple spionid species, as in the case of extant clams with shells parasitized by *Polydora brevipalpa*, *Dipolydora alborectalis*, and *Diploydora bidentata* simultaneously, each of which produces varying boring shapes.^20^ The fossilization of the Fezouata Shale boring traces as three-dimensional iron oxide replicates may have resulted from the pyritization of the organic matter contained within the original mudblisters, preserving the boring trace morphology after dissolution of the surrounding biomineralized shell. The fact that the boring traces are internal relative to the shell and that they show no evidence of mineralization or a hollow construction indicative of a secreted tube, allow us to discard alternative affinities for sabellid or serpulid polychetes as their producers.^1^ The new Fezouata Shale traces somewhat resemble the Paleozoic shell borings *Vermiforichnus* and *Caulostrepis*, which have been tentatively attributed to spionid polychetes.^5^ However, the taxonomic affinities of these traces are highly controversial^21^^,^^22^ and may not represent true spionid borings at all. The Fezouata traces further differ from the other ichnofossils based on their highly stereotyped question mark-like morphology and recurrent orientation within the host valve, and thus represent a different ichnotaxon altogether.

We identify a total of seven polydorid-like infestations in *Babinka* from the Fezouata Shale (Figures 1 and 2). The remarkable consistency in the boring trace morphology suggests a highly conserved expression of behavior in these Early Ordovician polychetes, an external physiological pressure leading to regulated burrowing such as paths of structural weakness in the host shell, or potentially a combination of both. It is also notable that while *Babinka* itself is a relatively rare taxon in the Fezouata Shale biota, roughly a third of all *Babinka* specimens show this highly distinctive evidence of parasitism. No other taxon shows evidence of a similar parasite in this deposit. Perhaps the most comparable taxonomic group in the Fezouata Shale to mollusk bivalves in terms of their broad autecology are the brachiopods, being another group of largely sessile, suspension-feeding, biomineralizers. Brachiopods are considerably more abundant in the MCZ collection, numbering approximately 372 specimens. However, none show any sign of parasitism by annelids or, indeed, any other organism, despite the known ability of modern spionids to parasitize brachiopods.^23^ This combination of high frequency and high specificity further supports the identification of the relationship as a legitimate case of symbiosis rather than a chance association.

The Fezouata Shale fossils indicate the presence of the family Spionidae by the late Tremadocian,^6^ extending their biostratigraphic range relative to their proposed Devonian body fossil record (although this fossil is not associated with a U-shaped burrow or mudblisters, and may in fact not represent a spionid^2^^,^^19^), and putative mid- to late-Ordovician trace fossil record (Figure 3C),^1^^,^^5^^,^^8^ indicating that parasitism had already evolved in this clade at the time. *Babinka* is considered to be a shallow-burrowing suspension feeder,^24^ thus the presence of spionid parasites suggests the ability of these taxa to exist in the meiofauna, or to otherwise disperse their larvae to buried bivalve shells. An infaunal host would presumably be disadvantageous for a spionid, as these groups are suspension feeders.^25^ However, modern spionids are parasites of both epibenthic and infaunal clams, albeit with a noted reduction in infestation frequency among infaunal conspecifics.^26^ This may indicate that the spionid parasites were able to either suspension feed on interstitial pore water as some modern interstitial animals do,^27^ or that *Babinka* was only a semi-infaunal to shallow-infaunal organism, as might be suggested based on the large active foot inferred from pedal attachment musculature.^24^ In this context, the consistent orientation of the spionid borings toward the margins of the shell may suggest a possible kleptoparasitic lifestyle using the currents generated by the bivalve host’s feeding, as has already been observed from Cambrian ectoparasites of brachiopods.^3^

The temporal shift in the origin of spionid polychetes is consistent with the emerging picture of annelid evolution including the presence of possible crown-group annelids by the early Cambrian.^28^ Thus, the Fezouata Shale fills an important stratigraphic gap for understanding the diversity and macroevolution of Paleozoic crown-group annelids (Figure 3C), since our understanding of this group is disproportionately informed by younger deposits with exceptional preservation such as the Silurian Herefordshire biota and the Carboniferous Mazon Creek.^8^

The spionid borings in *Babinka* capture the first record of parasitism in the Fezouata Shale biota.^6^ This discovery adds to the functional diversity of interspecific ecological interactions preserved in this deposit that so far include brachiopods attached to radiodont carapaces and conulariids attached to brachiopod valves.^29^ The fact that the Fezouata Shale has been studied for over 20 years^6^ highlights the rarity of this type of ecological information in the Paleozoic fossil record, even in sites with exceptional preservation, and emphasizes their value for reconstructing the emerging complexity of marine ecosystems during the initial stages of the Great Ordovician Biodiversification Event.

### Limitations of the study

Our study provides direct evidence of parasitism in Ordovician mollusks by annelids. However, the actual soft-tissues of the parasites themselves are not preserved. We can therefore only say that it is highly likely that the parasites were spionid annelids based on the high degree of similarity between their borings and those of extant analogs. We are also further limited in speculating the details of the life history strategy of the parasites given the lack of soft-tissue anatomy, but can reasonably speculate an ecology similar to their modern counterparts. Further study of Ordovician bivalves may reveal supporting data for our conclusions, and it is particularly important to continue detailed study of the entire Fezouata Shale biota, as the relative rarity of symbiotic relationships is far from fully explored.

## Resource availability

### Lead contact

Request for further information and resources should be directed to and will be fulfilled by the lead contact, Karma Nanglu (karma.nanglu@ucr.edu).

### Materials availability

Inquiries regarding access to figure material should be made to Javier Ortega-Hernández (jortegahernandez@fas.harvard.edu).

### Data and code availability

• A list of specimens with Museum of Comparative Zoology specimen numbers is included in Table 1, access to which can be provided by Javier Ortega-Hernández. Images of specimens can be provided by Javier Ortega-Hernández (jortegahernandez@fas.harvard.edu).
• This study did not involve any new original code.
• Any additional information required to reanalyze the data reported in this paper are available from the lead contact upon request. Tomographic datasets available in Dryad online repository (https://doi.org/10.5061/dryad.wwpzgmsxm).

## Acknowledgments

We acknowledge the key role of Mohamed “Ou Said” Ben Moula and the Ben Moula Family in the discovery, collection, and characterization of the studied fossil material and their contributions toward facilitating access the scientific study of the Fezouata Shale biota in Morocco. We thank Allison Daley (University of Lausanne), Peter Van Roy (Ghent University), and Stephen Pates (University College London) for their assistance recording field data in 2019. We thank MCZ Invertebrate Paleontology staff and researchers Jessica Cundiff and Mark Renczkowski for their help facilitating curation of the collections, Cyrus Green for tomographic scanning of the fossil specimens, Jared C. Richards for help with the identification and cataloging fossil specimens, and Bruno Becker Kerber for help with specimen photography. We thank V. Radashevsky for sharing original digital images of extant spionids. This work is funded by 10.13039/100006200NSF CAREER award no. 2047192 “Ecological turnover at the dawn of the Great Ordovician Biodiversification Event—Quantifying the Cambro-Ordovician transition through the lens of exceptional preservation.”

## Author contributions

Conceptualization, K.N. and J.O.-H.; methodology, M.E.W., S.R.L., and J.O.-H.; investigation, K.N. and J.O.-H.; resources, J.O.-H.; writing – original draft, K.N. and J.O.-H.; writing – review and editing, K.N. and J.O.-H.; visualization, M.E.W. and S.R.L.; funding acquisition, J.O.-H.

## Declaration of interests

The authors declare no competing interests.

## STAR★Methods

### Key resources table

**Table:** 

REAGENT or RESOURCE	SOURCE	IDENTIFIER
**Software and algorithms**		

Dragonfly version 2021.1	Comet Technologies Canada Inc.	N/A
ImageJ	LOCI, University of Wisconsin	N/A

### Experimental model and study participant details

All studied and figured fossil material is deposited at the Invertebrate Paleontology collections at the Museum of Comparative Zoology (MCZ.IP), Harvard University (Cambridge, USA). All studied specimens with trace fossils originate from the same sub-locality (30° 30’ 56” N, 5^o^ 49’ 34” W) of the Fezouata Shale in the vicinity of Zagora, Morocco. The co-occurrence of the studied *Babinka* specimens with the planktic graptolites *Kiaerograptus* and *Tetragraptus* in the same sub-locality indicate a late Tremadocian age as part of the *Araneograptus murrayi* biozone.

### Method details

Fossil material was photographed using a Nikon Z8 with a Nikkor Z MC 50mm lens under cross polarized light with Focus Shift Shooting and step width of 1, then stacked in ZereneStacker (zerenesystems.com/cms/home). Fossil specimens were scanned using a SkyScan1273 (Bruker Corporation) micro-computed tomography (micro-CT) scanner at the Museum of Comparative Zoology Digital Imaging Facilities. The slab containing MCZ.IP.202864 and MCZ.IP.202865 was scanned with a 1 mm copper filter with energy set at 130 kV/61 μA and an imaging pixel size of 44.41 μm. The slab containing MCZ.IP.202859 to MCZ.IP.202863 was scanned with a 2 mm copper filter with energy set at 130 kV/278 μA and an imaging pixel size of 35.63 μm. The scan files were reconstructed as cross-sectional tiff stacks in NRecon (Bruker Corporation) and visualized using Dragonfly software, Version 2021.1 for Windows. Comet Technologies Canada Inc., Montreal, Canada; software available at https://dragonfly.comet.tech/. Measurements of all fossils were performed using the software ImageJ.

### Quantification and statistical analysis

Angle measurements were calculated using the software ImageJ.
